# Isocitrate dehydrogenase mutation is associated with tumor location and magnetic resonance imaging characteristics in astrocytic neoplasms

**DOI:** 10.3892/ol.2014.2013

**Published:** 2014-03-28

**Authors:** SONGTAO QI, LEI YU, HEZHEN LI, YANGHUI OU, XIAOYU QIU, YANQING DING, HUIXIA HAN, XUELIN ZHANG

**Affiliations:** 1 Department of Neurosurgery, Nanfang Hospital, Southern Medical University, Guangzhou, Guangdong 510515, P.R. China; 2 Department of Pathology, Nanfang Hospital, Southern Medical University, Guangzhou, Guangdong 510515, P.R. China; 3 Department of Radiology, Nanfang Hospital, Southern Medical University, Guangzhou, Guangdong 510515, P.R. China

**Keywords:** isocitrate dehydrogenase, astrocytoma, tumor location, magnetic resonance imaging, prognosis

## Abstract

The molecular subsets of glioma behave in biologically distinct ways. The present study detected isocitrate dehydrogenase *(IDH*) 1 and *IDH2* mutations in glioma to analyze whether *IDH*-mutated gliomas are situated in certain preferential areas and to investigate their correlation with magnetic resonance imaging (MRI) characteristics. A series of 193 patients with astrocytic neoplasms (111 diffuse and 82 anaplastic astrocytomas), grouped according to prelabeled anatomical structures and the risk of surgery, were retrospectively reviewed for *IDH*1 and *IDH*2 mutations to compare the tumor location and MRI features. A total of 111 *IDH*1 mutations at codon 132 (57.5%) and six *IDH2* mutations at codon 172 (3.1%) were detected. The *IDH1/2* mutations were found to predict longer survival, independent of the histological type in this series of patients. The *IDH*-mutated gliomas were predominantly located in a single lobe, such as the frontal lobe, temporal lobe or cerebellum and rarely in the diencephalon or brain stem. Furthermore, according to the risk of surgery, the *IDH*-mutated tumors were rarely located in the high-risk regions of the brain, where surgery exhibits a high mortality rate intraoperatively and postoperatively. In addition, gliomas with *IDH* mutations were significantly more likely to exhibit a unilateral pattern of growth, sharp tumor margins, homogeneous signal intensity and less contrast enhancement on MRI. The results of the current study suggested that the prolonged survival of patients with *IDH*-mutated gliomas is primarily due to a less aggressive biological behavior according to tumor site and MRI features.

## Introduction

Gliomas are the most common type of tumor of the central nervous system in adults, with the glioma subtype, glioblastoma multiforme, the most common (1). A previous genome-wide mutational analysis of glioblastomas identified novel mutations in the isocitrate dehydrogenase (*IDH*) 1 gene (2). Further studies have demonstrated that *IDH1* mutations are present in 50–90% of cases of grade II and III astrocytoma and oligodendroglioma, but rarely present in primary glioblastoma or pilocytic astrocytoma (3–11). In addition to *IDH1* mutations, mutations in the homologous gene, *IDH2*, have also been identified in gliomas, but are much less common than the *IDH1* mutations (12,13).

Various retrospective and prospective studies have demonstrated that the *IDH* mutation is associated with longer survival in glioma patients (12–15). However, none of these studies have analyzed the correlation between *IDH* status and tumor location/magnetic resonance imaging (MRI) characteristics. Notably, different intracranial locations, such as functional or non-functional lobes, as well as the cortex or deep brain, are likely to cause variable difficulty levels for surgery, and thus, correspondingly influence the prognosis of patients. Furthermore, different presurgical MRI features, including well-defined or blurred interfaces, homogeneous or heterogeneous signal intensity and contrast enhancement level, are also likely to result in different extents of resection and residual tumor and therefore, different prognoses.

Since *IDH* mutation, tumor location and MRI features correlate with patient prognosis in glioma, the aim of the present study was to clarify the tumor location and MRI features of *IDH*-mutated gliomas to determine whether the prolonged survival of *IDH*-mutated patients is associated with tumor location and presurgical MRI features. Therefore, the radiological and genetic features of 193 patients affected by astrocytic neoplasms, with specific associations with *IDH* gene status were analyzed to address the aforementioned issues.

## Materials and methods

### Tumor samples

Patients were selected from a database of glioma patients treated at the Nanfang Hospital (Guangzhou, China) between January 2003 and December 2007. The eligibility criteria were as follows: Unequivocal pathological diagnosis according to the 2007 World Health Organization (WHO) criteria (16); availability of genetic analysis for *IDH1/2*; MRI scan at the time of the diagnosis or during the perioperative period; and ≥18 years old at the time of surgery. Following approval from the institutional review board, 193 evaluable patients with astrocytic neoplasms, consisting of 111 diffuse astrocytomas (DA) and 82 anaplastic astrocytomas (AA), were selected. The archival surgical specimens were enrolled after all patients had provided written informed consent allowing the molecular analysis of their tumor specimens. The pathology slides were reviewed by two neuropathologists and all tumor samples were confirmed by microscopic examination in which ≥70% of the visible cells in the section were tumor cells.

### Radiological assessment

All patients underwent MRI scanning prior to and following surgery (within 72 h) to evaluate the extent of surgery. All the MRI scans were independently reviewed by two neuroradiologists and a neurosurgeon blinded to the genetic alterations in the tumors. The tumor location and the following MRI features were evaluated qualitatively: Unilateral versus bilateral pattern of growth (tumors that traversed the corpus callosum to involve the opposite cerebral hemisphere were determined to be bilateral); sharp versus indistinct tumor margins; homogeneous versus heterogeneous signal intensity; absent or slight versus significant contrast enhancement; absent or moderate versus severe mass effect; and absent or moderate versus severe edema.

**Figure 1:**
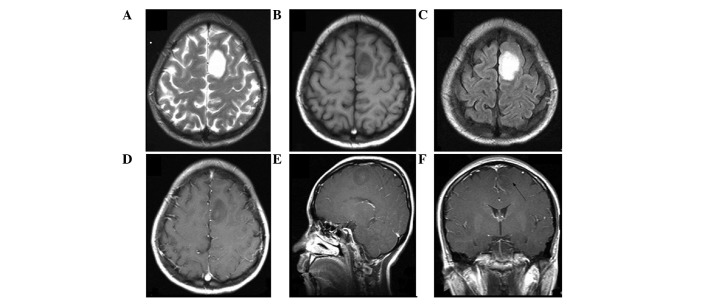
Patient with histological World Health Organization grade II diffuse astrocytoma and isocitrate dehydrogenase 1 mutation. Magnetic resonance imaging (A) T2-weighted, (B) T1-weighted, (C) FLAIR and (D) postcontrast T1-weighted axial as well as postcontrast T1-weighted (E) sagittal and (F) coronal (indicated by the black arrow) images demonstrated a lesion located in the posterior part of the superior frontal gyrus (non-functional region). The lesion (hyperintense on T2 images, hypointense on T1 images and hyperintense on FLAIR images with no postcontrast enhancement) showed well-demarcated, homogeneous high-signal intensity predominantly involving the white matter. No significant edema or mass effect were found adjacent to the cerebral falx. FLAIR, fluid-attenuated inversion recovery.

**Figure 2:**
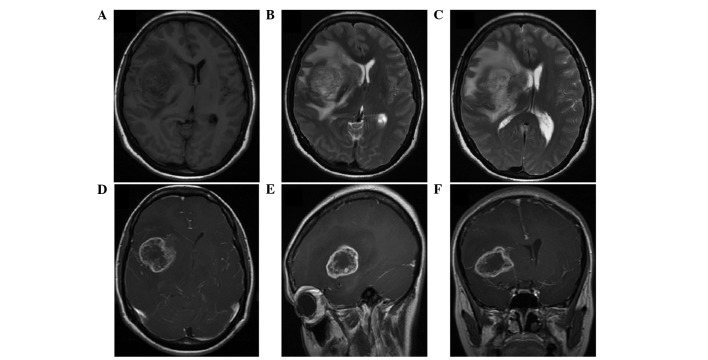
Patient with histological World Health Organization grade III anaplastic astrocytoma and no isocitrate dehydrogenase 1/2 mutation. Magnetic resonance imaging (A) T1-weighted (B and C) T2-weighted and (D) postcontrast T1-weighted axial as well as post-contrast T2-weighted (E) sagittal and (F) coronal images revealed an ill-defined insular mass, measuring 3.2×2.9 cm, near the anterior limb of the internal capsule (functional region). Following contrast administration, an intense, irregular enhancement was recognized and a significant mass effect was observed adjacent to the basal ganglia with evident edema.

**Figure 3:**
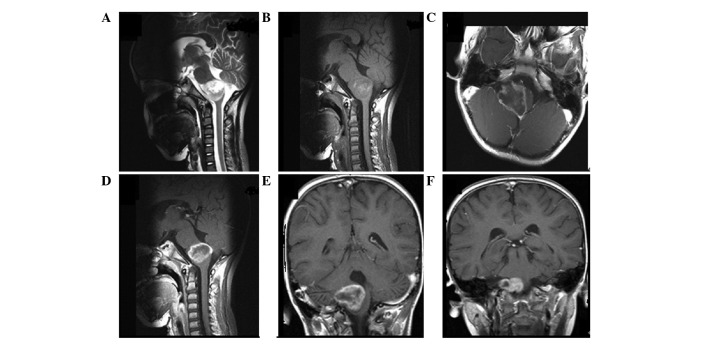
Patient with histological World Health Organization grade III anaplastic astrocytoma and no isocitrate dehydrogenase 1/2 mutation. Magnetic resonance imaging (A) T2-weighted, (B) T1-weighted and (C) postcontrast T1-weighted axial as well as post-contrast T1-weighted (D) sagittal and (E and F) coronal images demonstrated an ill-defined mass severely invading the medulla oblongata (high-risk region). The lesion showed heterogeneous T2 hyperintense and T1 iso-hyperintense signals with apparent enhancement following the contrast administration and a significant mass effect was observed without edema.

To define the tumor location, the following grouping methods were considered. Tumor location defined using the following prelabeled anatomical structures in the brain: i) Frontal, temporal, parietal, occipital lobes and cerebellum; ii) insula, diencephalon, basal ganglia; and iii) brain stem. Tumor location divided into the following three groups according to the risk of surgery: Group I, high-risk regions (such as the hypothalamus, midbrain and medulla oblongata); group II, functional regions (for example the primary sensorimotor area, supplementary motor area, internal capsule and basal ganglia); and group III, non-functional regions (remote from high-risk regions and functional regions) at the time of diagnosis (Figs. 1–3). Regardless of the grouping methods, the tumor location was typically determined by joint analysis of sagittal, coronal and axial MRI sequences. In addition, the site of origin and location of the epicenter of the tumor were considered simultaneously to determine the tumor location.

### IDH1/2 mutation analysis

Genomic DNA was extracted from the formalin-fixed, paraffin-embedded tissues using the QIAamp DNA mini kit (Qiagen, Hilden, Germany) according to the manufacturer’s instructions. *IDH1* and *IDH2* alterations characterized by mutational hotspots at codons R132 and R172, respectively, were assessed by high resolution melting (HRM) analysis (17) and direct sequencing, which were performed using the ABI PRISM 3730xl DNA analyzer (Applied Biosystems, Carlsbad, CA, USA). The polymerase chain reaction (PCR) products generated after HRM were sequenced directly following purification with the QIAquick PCR purification kit (Qiagen, Valencia, CA, USA). The PCR primers for mutations were designed using Primer Express software version 3.0 (Applied Biosystems). The primers sequences used were as follows: Forward, 5′-CGGTCTTCAGAGAAGCCATT-3′ and reverse, 5′-GCAAAATCACATTATTGCCAAC-3′ for *IDH1*; and forward, 5′-CCAAGCCCATCACCATTG-3′ and reverse, 5′-ACTGGAGCTCCTCGCCTAGC-3′ for *IDH2*. The PCR and HRM analyses were performed in a single run using the LightCycler 480 instrument (Roche Diagnostics GmbH, Penzberg, Germany) in a reaction mixture to discriminate between the wild-type and mutant DNA. Samples exhibiting conflicting HRM and direct sequencing results were retested and only the HRM-positive samples confirmed by direct sequencing were considered mutated.

### Statistical analysis

Statistical analyses were conducted using SPSS version 13.0 (SPSS, Inc., Chicago, IL, USA). The χ^2^ test (or Fisher’s exact test when one subgroup was n<5) was used to determine the significance of associations. The Bonferroni test was used for multiple comparisons and the independent samples t-test was used to compare data acquired in each group for patients age. Progression-free survival (PFS) and overall survival (OS) were used to analyze the prognostic impact of *IDH1/2* mutations. PFS was calculated from the initial surgery until the first unequivocal clinical or radiological sign of progressive disease, or the last follow-up (for censored cases) and OS was defined as the time between the initial surgery and mortality, or the last follow-up (for censored cases). The survival distributions were estimated using the Kaplan-Meier method and compared among the patient subsets using log-rank tests. All statistical tests were two-sided and P<0.05 was considered to indicate a statistically significant difference. Patients who succumbed to the disease within two months for DA and one month for AA following surgery were excluded from the analysis to avoid the inclusion of cases in which mortality may have been attributable to surgical complications.

## Results

### IDH1 and IDH2 mutations

A total of 193 astrocytic neoplasms, which fulfilled the inclusion criteria, were retrospectively analyzed. *IDH1* and *IDH*2 mutations were identified in 57.5% (111/193) and 3.1% (6/193) of the patients, respectively. All *IDH1* mutations were located at the amino acid residue 132, of which 102 were R132H (G395A Arg132His), six were R132C (C394T Arg132Cys) and three were R132S (C394A Arg132Ser) mutations, whereas all *IDH*2 mutations were observed at the amino acid residue 132, of which four were R172K (G515A Arg172Lys) and two were R172M (G515T Arg172Met) mutations. As previously reported (18,19), these two mutations are mutually exclusive as observed in 100% of cases in this series, suggesting that they are involved in similar tumorigenesis pathways. Therefore, in the current statistical analysis the *IDH1* and *IDH*2 mutations were grouped together. The main clinical characteristics of the patients are summarized in Table I.

### IDH1/2 mutations predict longer survival

The mean follow-up of the patients was 63.3 months (range, 15–101 months) and as indicated in Table I, a significant difference was indicated between patients with and without the *IDH1/2* mutations and PFS (P<0.001) and OS (P<0.001), independent of WHO grade. The prognostic impact of the *IDH1/2* mutations in the DA and AA subgroups was also investigated and the *IDH1/2* mutations were found to significantly correlate with increased survival in the subgroups (data not shown).

### Correlation between IDH status and tumor location

In this series of astrocytic neoplasms, a statistically significant correlation was identified between *IDH* status (*IDH*-mutated versus *IDH* wild-type) and tumor location. In addition, the lobar distribution of the neoplasms was analyzed in all patients and the *IDH*-mutated tumors were more frequently located in a single lobe, such as the frontal lobe, temporal lobe or cerebellum, whereas the *IDH* wild-type tumors were predominantly located in combined lobes, such as the diencephalon or brain stem (P<0.001; χ^2^ test; Table II). According to an additional grouping method, the *IDH*-mutated tumors were rarely located in the high-risk regions of the brain and more frequently located in the non-functional and functional regions (P<0.001; χ^2^ test; Table III). Although *IDH*-mutated tumors invaded high-risk regions less frequently than the non-functional and functional regions, no difference was observed in the frequency of *IDH1/2* mutations between the non-functional and functional regions (Table III).

### Correlation between IDH status and MRI characteristics

The correlation between the preoperative MRI features and *IDH* status in all patients and the different histological subgroups was also investigated. The histopathological *IDH* status and imaging features are summarized in Table IV. As illustrated in Table IV, the gliomas with *IDH* mutations were significantly more likely to exhibit a unilateral pattern of growth, sharp tumor margins, homogeneous signal intensity and less contrast enhancement in the DA and AA subgroups. Analysis of all the patients also confirmed this difference, however, no significant correlation was identified between the remaining MRI features (mass effect and edema) and *IDH* status in subgroups and all patients.

## Discussion

The tumor location and MRI features of glioma are important indicators of prognosis and the tumor location determines the resectability of the glioma; tumors located in the critical areas of the brain are typically non-resectable whereas those located in the non-functional regions may undergo en bloc extended resection to prolong survival. MRI findings which are suggestive of high-grade gliomas, including a bilateral pattern of growth, undefined margins, mixed signal intensity and significant enhancement reflect increased ‘invasiveness’ and high malignancy of the glioma, which often indicate unfavorable outcomes. Notably, increasing age has classically been associated with a poor prognosis in gliomas and patients with *IDH* wild-type tumors are significantly older than those with *IDH*-mutated tumors, which is consistent with the results of the present study (Table I) (2,3,5,7). However, few studies have analyzed the correlation between *IDH* status and tumor location/MRI characteristics.

Recently, Metellus *et al* (20) reported that *IDH* wild-type WHO grade II gliomas are preferentially located in the fronto-temporo-insular region and exhibit a greater volume and therefore, require a reduced extent of surgery and demonstrate an infiltrative pattern on MRI. This is the only study to identify a statistically significant correlation between *IDH* status and specific brain subregions of tumor locations. By contrast, patients with oligodendroglial tumors were excluded in the current study as astrocytoma and oligodendroglioma exhibit different biological behaviors and clinical features (21–23). In addition, the correlation between *IDH* status and tumor location was analyzed in the present study based on a larger sample size and more comprehensive grouping methods, determined not only by prelabeled anatomical structures but also by the risk of surgery. The *IDH*-mutated gliomas were rarely found to locate in the high-risk regions of brain, such as the diencephalon or brain stem, where surgical resection is limited and exhibits a high mortality rate intraoperatively and postoperatively. The *IDH*-mutated gliomas were instead preferentially found to locate in the functional or non-functional regions, particularly the frontal and temporal lobes, where tumors can be removed easily. These results were supported by the observation that the extent of surgery (gross total resection and subtotal resection) was significantly higher in *IDH*-mutated gliomas compared with *IDH* wild-type gliomas (P=0.002; Table I).

This correlation between genotype and tumor site has also been reported in oligodendroglial neoplasms and glioblastoma. Furthermore, in a pioneering study by Zlatescu *et al* (24), the anaplastic oligodendrogliomas located in the frontal, parietal and occipital lobes were significantly more likely to harbor the 1p19q codeletion than tumors arising in the temporal lobe, insula and diencephalon. More recent studies have identified a significant correlation between the 1p19q codeletion and tumor location in oligodendrogliomas or oligoastrocytomas (25–29). However, conflicting results have been reported regarding the correlation between O6-methylguanine DNA methyltransferase (MGMT) promoter methylation status and tumor location in glioblastoma. Eoli *et al* (30) also demonstrated that tumors with MGMT promoter methylation were more frequently located in the parietal and occipital lobes, whereas tumors without were frequently located in the temporal lobes, however, other studies have not identified such a correlation (31). In addition, astrocytomas have not been found to exhibit different frequencies of the 1p19q codeletion or tumor protein p53 mutations with respect to tumor location (25,26).

Significant associations have also been reported between MRI characteristics and genotype in oligodendrogliomas, oligoastrocytomas or glioblastomas (20,29,31–37), however, *IDH* mutation status was not addressed in these studies, with the exception of that by Metellus *et al*. Consistent with the results reported by Metellus *et al* (20), the present study also revealed that *IDH*-mutated gliomas are significantly more likely to exhibit sharp tumor margins. Furthermore, it was observed that *IDH*-mutated tumors tend to exhibit a higher incidence of unilateral pattern of growth, homogeneous signal intensity and less contrast enhancement. Therefore, the *IDH*-mutated tumors exhibit less invasiveness when compared with *IDH* wild-type tumors in MRI.

It is evident that the presence of *IDH* mutations is of major prognostic significance for patient outcome in gliomas and the current study found a marked correlation between the *IDH* mutations and OS (independent of WHO grade) in this series of patients. However, at present, the underlying mechanism of *IDH* mutations in tumorigenesis and prognostic significance remains unclear. A prospective randomized European Organization for Research and Treatment of Cancer study 26951 reported by van den Bent *et al* (38), revealed no indication that the presence of the *IDH1* mutation predicts the outcome to adjuvant procarbazine, 1-(2-chloroethyl)-3-cyclohexyl-nitrosourea, and vincristine chemotherapy. In an additional retrospective report on temozolomide chemotherapy in progressive low-grade astrocytoma, no correlation was identified between outcome and the *IDH* mutations (39). These results suggested that the improved survival observed in *IDH1*-mutated tumors is primarily due to a less aggressive biological behavior and not due to an improved outcome for chemotherapy treatment (38).

In conclusion, the current study investigated the correlation between *IDH* status and tumor location, as well as MRI characteristics in astrocytic neoplasms and revealed that the prolonged survival of patients with *IDH1*-mutated tumors is primarily due to a less aggressive biological behavior from the perspective of tumor site and MRI features.

## Figures and Tables

**Table I:** Main clinical characteristics.

Variables	Total population	IDH1/2 mutations	IDH wild-type	P-value
n, (%)	193 (100)	117 (60.6)	76 (39.4)	
Age, years				<0.001
Median	36.5	32.7	42.5	
Range	18–72	19–67	18–72	
Gender, n (%)				0.768
Male	109 (100)	65 (59.6)	44 (40.4)	
Female	84 (100)	52 (61.9)	32 (38.1)	
KPS at diagnosis, n (%)				0.289
≥80	119 (100)	76 (63.9)	43 (36.1)	
<80	74 (100)	41 (55.4)	33 (44.6)	
Histology (WHO grade), n (%)				0.181
DA (II)	111 (100)	72 (64.9)	39( 35.1)	
AA (III)	82 (100)	45 (54.9)	37 (45.1)	
Extent of surgery, n (%)				0.002
Biopsy/PR	89 (100)	43 (48.3)	46 (51.7)	
STR/GTR	104 (100)	74 (71.2)	30 (28.8)	
PFS, months				<0.001
Median	45.8	56.7	34.4	
95% CI	42.0–49.6	51.1–62.3	29.1–39.7	
OS, months				<0.001
Median	71.3	84.3	57.3	
95% CI	66.1–76.5	79.0–89.6	46.2–68.4	

AA, anaplastic astrocytoma; DA, diffuse astrocytoma; GTR, gross total resection; IDH1/2, isocitrate dehydrogenase 1/2 mutation; KPS, Karnofsky performance status; OS, overall survival; PR, partial resection; PFS, progression-free survival; STR, subtotal resection; WHO, World Health Organization; CI, confidence interval.

**Table II:** Analyzing the frequency of *IDH1/2* mutations and tumor location according to anatomical structures.

Histology	F, n	T, n	P or O, n	Multilobes, n	I or BG, n	D or BS, n	CB, n
DA							
n	45	9	9	11	14	12	11
IDH mutation	38	7	8	3	6	1	9
IDH wild-type	7	2	1	8	8	11	2
AA							
n	21	16	8	17	4	14	2
IDH mutation	15	13	4	5	3	4	1
IDH wild-type	6	3	4	12	1	10	1
Overall							
n	66	25	17	28	18	26	13
IDH mutation	53	20	12	8	9	5	10
IDH wild-type	13	5	5	20	9	21	3

AA, anaplastic astrocytoma; BG, basal ganglia; BS, brain stem; CB, cerebellum; D, diencephalon; DA, diffuse astrocytoma; F, frontal lobe; IDH, isocitrate dehydrogenase; I, insular lobe; multilobes, combined lobes; O, occipital lobe; P, parietal lobe; T, temporal lobe.

**Table III:** Analyzing the frequency of the *IDH1/2* mutations and tumor location according to the risk of surgery.

Variables	Group I vs. II	Group I vs. III	Group II vs. III
Overall, n (%)	37 vs. 63	37 vs. 93	63 vs. 93
IDH mutation	11 (29.7) vs. 39 (61.9)	11 (29.7) vs. 67 (72.0)	39 (61.9) vs. 67 (72.0)
IDH wild-type	26 (70.3) vs. 24 (38.1)	26 (70.3) vs. 26 (28.0)	24 (38.1) vs. 26 (28.0)
χ^2^ test	9.653	19.746	1.773
P-value^a^	0.003	<0.001	0.222

a Bonferroni test was performed for multiple comparisons and Bonferroni-corrected P<0.05/3 was considered to indicate a statistically singificant difference.

Groups I, high-risk; II, function regions; and III, non-functional regions. IDH, isocitrate deyhydrogenase.

**Table IV:** Analyzing the frequency of *IDH1/2* mutations and different MRI features of gliomas.

MRI features	All^a^, n (%)	P-value	DA^a^, n (%)	P-value	AA^a^, n (%)	P-value
Pattern of growth		<0.001		0.007		0.001
Unilateral	116/178 (65.2)		71/104 (68.3)		45/74 (60.8)	
Bilateral	1/15 (6.7)		1/7 (14.3)		0/8 (0.0)	
Tumor margins		<0.001		0.001		0.012
Sharp	66/85 (77.6)		44/55 (80.0)		22/30 (73.3)	
Indistinct	51/108 (47.2)		28/56 (50.0)		23/52 (44.2)	
Tumor signal intensity		<0.001		0.003		<0.001
Homogeneous	70/89 (78.7)		45/58 (77.6)		25/31 (80.6)	
Heterogeneous	47/104 (45.2)		27/53 (50.9)		20/51 (39.2)	
Contrast enhancement		<0.001		0.001		0.003
Absent or slight	74/97 (76.3)		47/60 (78.3)		27/37 (73.0)	
Significant	43/96 (44.8)		25/51 (49.0)		18/45 (40.0)	
Mass effect		0.654		0.320		0.216
Absent or moderate	47/75 (62.7)		38/54 (70.4)		9/21 (42.9)	
Severe	70/118 (59.3)		34/57 (59.6)		36/61 (59.0)	
Edema		0.181		0.533		0.375
Absent or moderate	71/109 (65.1)		49/73 (67.1)		22/36 (61.1)	
Severe	46/84 (54.8)		23/38 (60.5)		23/46 (50.0)	

a Number of mutated samples/total number of samples of given type grouped by the different MRI features of gliomas.

AA, anaplastic astrocytoma; DA, diffuse astrocytoma; MRI, magnetic resonance imaging; IDH, isocitrate dehydrogenase.
